# Research and Implementation of a Demodulation Switch Signal Phase Alignment System in Dynamic Environments

**DOI:** 10.3390/s23229144

**Published:** 2023-11-13

**Authors:** Ke Xue, Tao Yu, Yanlin Sui, Yongkun Chen, Longqi Wang, Zhi Wang, Jun Zhou, Yuzhu Chen, Xin Liu

**Affiliations:** 1 Changchun Institute of Optics, Fine Mechanics and Physics, Chinese Academy of Sciences, Changchun 130033, China; xuekeciomp@163.com (K.X.);; 2 School of Fundamental Physics and Mathematical Sciences, Hangzhou Institute for Advanced Study, University of Chinese Academy of Sciences, Hangzhou 310024, China

**Keywords:** inertial sensors, capacitive sensing, phase alignment

## Abstract

In the space gravitational wave detection mission, inertial sensors play the role of providing an inertial reference for the laser interferometric measurement system. Among them, the capacitance sensor serves as the core key technology of the inertial sensor, used to measure the relative position of the test mass (TM) in the electrode cage. The capacitance sensor utilizes synchronous demodulation technology to extract signals from the AC induction signal. When the phase of the demodulation switch signal is aligned, the synchronous demodulator can most effectively filter out noise, thus directly influencing the performance of the capacitance sensor. However, since the TM is in a suspended state, the information read by the capacitance sensor is dynamic, which increases the difficulty of demodulation phase alignment. In light of this, a method is proposed for achieving the phase alignment of the demodulation switch signal in a dynamic environment. This is accomplished by adjusting the phase of the demodulation switch signal, and subsequently computing the phase difference between the AC induction signal and the demodulation switch signal. At the same time, a measurement and evaluation method for phase deviation is also proposed. Ultimately, an automatic phase alignment system for the demodulation switch signal in dynamic environments is successfully implemented on an FPGA platform, and tests are conducted on a hexapod PI console platform to simulate dynamic environments. The experimental results demonstrate that the system accurately achieves phase alignment in the static environment, with a phase deviation of 0.1394 rad. In the simulated dynamic environment, the phase deviation is 0.1395 rad.

## 1. Introduction

Inertial sensors are the central payload in space-based gravitational wave detection missions, providing an inertial reference for the laser interferometric measurement system. They play a pivotal role in missions like LISA, Taiji, and Tianqin [1,2,3,4,5], directly influencing the sensitivity of gravitational wave detection. Capacitive sensing is a critical technology for inertial sensors. Its primary function is to measure the capacitance variation caused by the motion of the test mass (TM) within the electrode cage, and its measurement accuracy is directly related to the sensor’s resolution [6]. Capacitive sensing uses a synchronous demodulator to extract the position information of the TM from the AC induction signal [7]. To improve the signal-to-noise ratio of capacitive sensing, synchronous demodulation uses appropriate switching signals to lock the AC induction signal frequency, effectively eliminating noise. However, the phase difference between the AC induction signal and the demodulation switch signal directly affects the performance of the demodulator. A slight phase difference can lead to some noise not being eliminated, thereby reducing the capacitance resolution of the capacitive sensing circuit [8,9]. Meanwhile, the TM is in a suspended state, causing dynamic changes in the position information read by the capacitive sensor. This increases the difficulty of phase alignment for demodulation.

A synchronous demodulator, also known as a locked-in amplifier, plays a core role in capacitive sensing. It comprises a multiplier and a low-pass filter. The principle involves multiplying the received AC induction signal with the demodulation switch signal to extract the necessary amplitude information [10]. To prevent distortion of the demodulation output, it is crucial to ensure that the AC induction signal aligns in both frequency and phase with the demodulation switch signal. Synchronous demodulators can be categorized into two types based on their circuit structure. The first type is the analog structure [11,12], as detailed in references [6,7,13], which employs a switched structure to achieve the demodulation function. This structure can achieve a reduction in 1/f noise, thereby enhancing the performance of capacitive sensors. The other type is a digital structure [14], commonly implemented using DSP or FPFA [15], which can significantly enhance the processing speed and accuracy of the system. This aids in extracting information from weak modulated signals [16]. However, the analog demodulator structure commonly used in inertial sensors cannot achieve real-time demodulation, so it is necessary to align the phase of the demodulation switch signal. At the same time, the phase of the demodulation switch signal needs to be aligned before the inertial sensor switches the working mode.

However, the precise phase alignment of demodulated switch signals in dynamic environments poses a challenge, and when phase alignment is not achieved, it can impact the signal-to-noise ratio (SNR) of capacitive sensing. Hence, there is a need to develop a phase alignment system capable of achieving accurate phase alignment in dynamic environments. This system can effectively reduce interference in dynamic environments, and ensures precise phase alignment, rendering it suitable for capacitive sensing measurements under dynamic conditions in both ground-based and space-based experiments.

Simultaneously, phase alignment technology is extensively employed in precision measurements [17,18] and can be categorized into two structures. One approach [19,20,21] employs a dual-channel method for phase alignment, where a pair of orthogonal reference signals is multiplied by the input signal to calculate the current phase difference and ultimately achieve phase alignment. While this method allows for a precise measurement of resistance and capacitance, it does lead to an increase in the circuit’s power consumption. Another approach [22,23,24,25] utilizes a single-channel method for phase alignment, by a π/2 phase shift of the reference signal. This ultimately allows for the determination of the phase difference between the reference signal and input signal, achieving phase alignment. Nevertheless, methods of this kind are prone to noise interference, resulting in diminished performance.

However, when inertial sensors operate in space, the TM is suspended within an electrode cage, and its position experiences dynamic changes. This presents a challenging condition for phase alignment. During ground-based testing of inertial sensors, the LISA team [26] designed a low-pass filter capable of filtering out resonance frequency components of the TM, greatly reducing the impact of noise on phase alignment. In [27,28], a low-pass filter (LPF) is implemented on an FPGA platform and incorporated into the demodulation circuit. This approach showcases impressive noise suppression capabilities and outstanding dynamic performance.

In response to the dynamic phase alignment issue of the demodulation module for the moving TM, this paper designs a phase alignment system. Taking into account the energy consumption and reliability of capacitive sensing in space, the phase alignment system employs a single-channel structure. It adjusts the demodulation switch signal, computes the phase difference between the AC induction signal and the demodulation switch signal, and aligns the phase. Eventually, on the FPGA platform, the automatic phase alignment of the demodulation switch signal is successfully implemented in dynamic environments.

The above method is not only applicable to the development of space inertial sensors but also finds application in various fields including accelerometers and weak signal detection.

In this paper, our main contributions are as follows:

(1) This paper introduces phase alignment system in dynamic environments, to solve the difficulty of phase adjustment. The system can determine the phase difference by shifting the phase of the demodulation switch signal by π/2, thereby achieving a precise phase alignment of the signal.
(2) We create a simulated dynamic environment to test the phase alignment system. The experimental results demonstrate that the system can accurately align the phase of the demodulation switch signal even in the presence of dynamic environmental interference.
(3) This paper introduces a method for measuring phase deviation. Through adjusting the amplitude of the injection voltage, the phase deviation of the demodulation switch signal is ultimately fitted.

This paper is structured as follows. Section 2 introduces the working principle of capacitive sensing and the operation of synchronous demodulators in dynamic environments. Section 3 introduces the design of the phase alignment system of the demodulation switch. Section 4 discusses the measurement results of this system in both static and dynamic environments. The final section offers a comprehensive summary of the paper.

## 2. Synchronous Demodulators in Dynamic Environments

### 2.1. Working Principle of Capacitor Sensors

**Figure 1:**
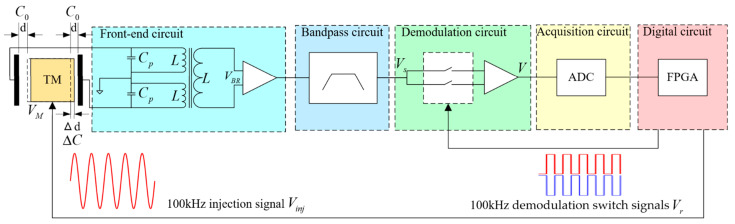
The working principle of single-channel capacitive sensing circuit (including TM, front-end circuit, bandpass circuit, demodulation circuit, acquisition circuit, and digital circuit).

The capacitive sensing circuit adopts the bridge-detection circuit scheme, and its principle is shown in Figure 1. For simplicity, only one direction is depicted to measure slight changes in capacitance resulting from small displacement. The TM is located in the center between the two electrodes; that is, the distance from the electrode to TM is d and the effective area of the electrode plate is S.

(1) $$C_{0}=C_{1}=C_{2}=\frac{\varepsilon_{r}\varepsilon_{0}S}{d}$$

where $\varepsilon_{0}$ is the vacuum permittivity and $\varepsilon_{r}$ is the relative permittivity. And when TM shifts Δd in the electrode cage, it causes a change of differential capacitance to ΔC.

(2) $$\Delta C=C_{1}-C_{2}={2\varepsilon }_{r}\varepsilon_{0}S\frac{\Delta d}{d^{2}}$$

The 100 kHz AC injection voltage signal $V_{inj}$ generated by the digital circuit is applied to the electrode, causing the injection voltage $V_{M}$ to be generated at the TM surface and causing a resonance at the 100 kHz frequency within the front-end circuit. The output voltage of the capacitive-inductive resonant bridge can be described by Equation (3), where ω represents the resonant frequency, $C_{p}$ is the sensing bridge tuning capacitance, and L denotes inductance [7].

(3) $$V_{BR}=V_{M}\frac{-\omega^{2}L}{1-{2\omega }^{2}L\left(C_{0}+C_{p}\right)}$$

Subsequently, the resulting AC induction signal $V_{S}$ is filtered through a bandpass circuit. The filtered AC induction signal is then fed into the synchronous demodulation circuit. Meanwhile, a pair of opposing 100 kHz demodulation switch signals, generated by the digital circuit, controls the demodulation switch. Under the control of the demodulation switch signals, the AC induction signal has its DC component extracted by the synchronous demodulation circuit. Subsequently, it is collected by the acquisition circuit and ultimately fed into the digital circuit for processing.

To build upon comprehension of the capacitance sensing circuit, it is crucial to underscore the pivotal role played by the phase of the demodulation switch signal in synchronous demodulation performance. This paper centers on investigating the phase alignment of the demodulation switch signal in dynamic environments and its practical implementation using FPGA technology.

### 2.2. Working Principle of Synchronous Demodulations

**Figure 2:**
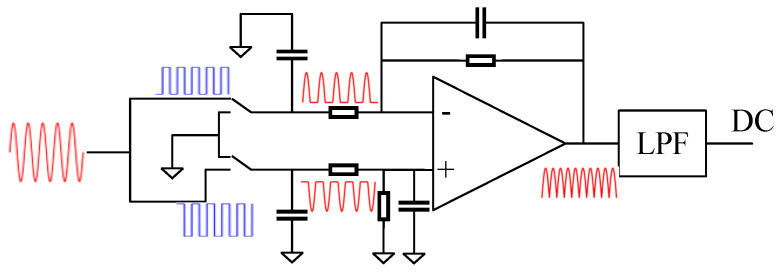
Demodulation circuit structure (including switch synchronous demodulation structure and LPF).

In this paper, the synchronous demodulation circuit plays an important role in capacitive sensing. As shown in Figure 2, a switch demodulation scheme is adopted. The AC induction signal is split into two inputs into the switching demodulation circuit, controlled by a pair of demodulation switching signals with the same frequency but opposite phase. After demodulation switch processing, a pair of phase-opposite half-wave signals is obtained. Then, the pair of phase-opposite half-wave signals is combined by an adder to obtain a full-wave rectified signal. Ultimately, the low-pass filter processes the output AC induction signal to extract amplitude information.

In the demodulation circuit, the input AC induction signal is

(4) $$V_{s}\left(t\right)=A\cos\left(\omega_{0}t+\theta \right)$$

where A is the amplitude of AC induction signal, $\omega_{0}$ is the signal’s frequency, and θ is the phase difference between the AC induction signal and the demodulation switch signal.

The demodulation switch signal, denoted as $V_{r}\left(t\right)$, is a square-wave signal with an amplitude of ±1. Additionally, its frequency matches that of the AC induction signal. And its Fourier series is Equation (5), where $a_{0}$ is the DC component, $a_{m}$ is the Fourier coefficient of its cosine component, and $b_{m}$ is the Fourier coefficient of its sine component. $V_{r}\left(t\right)$ is an even function whose mean is zero, the $a_{0}$ and $b_{m}$ are zero, and the Fourier coefficient $a_{m}$ of its cosine component is Equation (6).

(5) $$V_{r}\left(t\right)=a_{0}+\sum_{m=1}^{\infty }a_{m}\cos m\omega_{0}t+\sum_{m=1}^{\infty }b_{m}\sin m\omega_{0}t$$

(6) $$a_{m}=\frac{1}{\pi }\int_{-\pi }^{\pi }V_{r}\left(t\right)\cos\left(m\omega_{0}t\right)d\left(\omega_{0}t\right)=\frac{1}{\pi }\left(\int_{-\pi /2}^{\pi /2}\cos\left(m\omega_{0}t\right)d\left(\omega_{0}t\right)-\int_{-\pi }^{-\pi /2}\cos\left(m\omega_{0}t\right)d\left(\omega_{0}t\right)-\int_{\pi /2}^{\pi }\cos\left(m\omega_{0}t\right)d\left(\omega_{0}t\right)\right)=\frac{4}{m\pi }\sin\left(\frac{m\pi }{2}\right)$$

when m is even, $\sin\left(m\pi /2\right)=0$, and for odd m, $\sin\left(m\pi /2\right)=\pm 1$. Let m=2n−1, where n is a positive integer; then, $a_{m}$ is Equation (7). And the Fourier series of the demodulation switch signal is Equation (8).

(7) $$a_{m}=\frac{4}{\pi }\frac{{\left(-1\right)}^{n+1}}{2n-1}$$

(8) $$V_{r}\left(t\right)=\frac{4}{\pi }\sum_{n=1}^{\infty }\frac{{\left(-1\right)}^{n+1}}{2n-1}\cos\left(\left(2n-1\right)\omega_{0}t\right)$$

The output of the demodulation switch circuit is derived by multiplying the AC induction signal with the demodulation switch signal:

(9) $$V\left(t\right)=V_{s}\left(t\right)V_{r}\left(t\right)=\frac{2A}{\pi }\sum_{n=1}^{\infty }\frac{{\left(-1\right)}^{n+1}}{2n-1}\cos\left(\left(2n-2\right)\omega_{0}t-\theta \right)+\frac{2A}{\pi }\sum_{n=1}^{\infty }\frac{{\left(-1\right)}^{n+1}}{2n-1}\cos\left(2n\omega_{0}t+\theta \right)$$

After processing through the low-pass filter to eliminate the high-frequency component, the output of synchronous demodulation is denoted as

(10) $$V\left(t\right)=\frac{2A}{\pi }\cos\theta$$

Clearly, when the phase difference is zero, synchronous demodulation yields the highest output, leading to the highest resolution in capacitive sensing. Simultaneously, synchronous demodulation effectively averages out other (noise) frequencies, enhancing the overall performance to its optimum.

### 2.3. Effect of Dynamic Environments on Phase Alignment

When the inertial sensor operates in space, the TM is suspended, and its position information is dynamically changing. Capacitive sensors are employed to measure the TM’s position information, leading to dynamic changes in the input signal of the synchronous demodulator. As Equation (4) shows, the output of synchronous demodulation is expected to follow Equation (11), with its amplitude varying over time. Likewise, the output of synchronous demodulation corresponds to Equation (12), inducing significant interference in the phase alignment of the demodulation switch signal.

(11) $$V_{s}\left(t\right)=A\left(t\right)\cos\left(\omega_{0}t+\theta \right)$$

(12) $$V\left(t\right)=\frac{2A\left(t\right)}{\pi }\cos\theta$$

**Figure 3:**
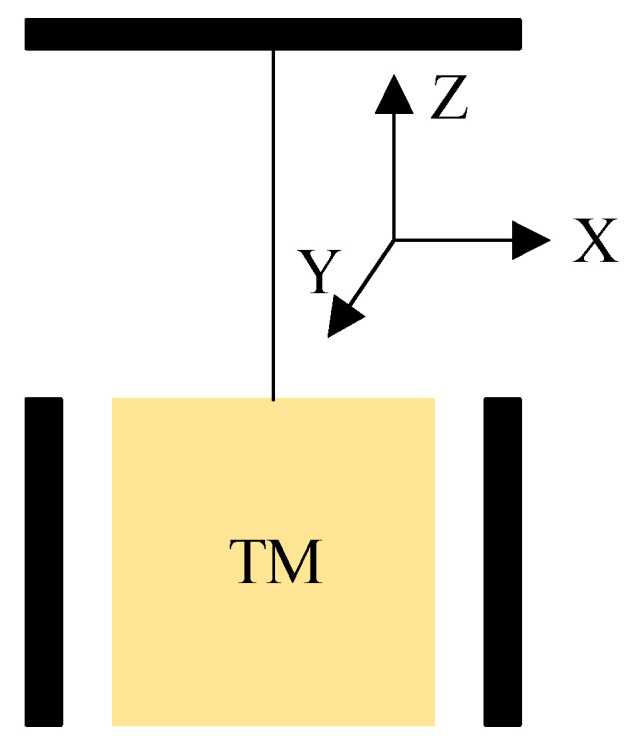
Simple diagram of torsion pendulums (TM is suspended by the tungsten wire and can swing horizontally along the *X*-axis and *Y*-axis, and rotate around the *Z*-axis).

This phenomenon is particularly evident in ground-based test experiments involving inertial sensors. Typically, ground experiments with inertial sensors are conducted using torsion pendulums. Illustrated in Figure 3, this study employs tungsten wire for suspending the TM within the electrode cage. Subsequently, it captures the positional data of the TM using a capacitive sensor. The natural frequency of its oscillation is represented by Equation (13), with l denoting the length of the tungsten wire, $l^{\prime }$ indicating the distance from the suspension point of the tungsten wire to its center of gravity on the TM, and g standing for the gravitational constant.

(13) $$f_{SW}=\frac{1}{2\pi }\sqrt{\frac{g}{l+l^{\prime }}}$$

**Figure 4:**
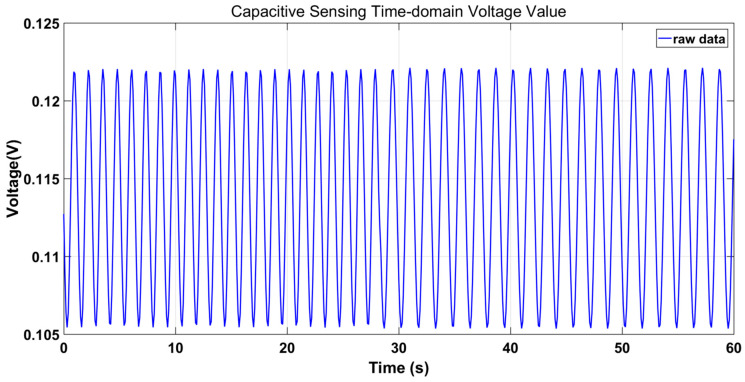
Capacitance sensing measures the time-domain voltage value of the TM for one minute along the *X*-axis.

Referring to the basic torsion pendulums set up in the laboratory, the TM operates in a dynamic environment, and its positional data along the *X*-axis are acquired via the capacitive sensor. As depicted in Figure 4, when the TM is in dynamic environments, the reading of the capacitance sensor changes dynamically. It can be seen that the phase alignment of demodulated switch signals is very challenging in dynamic environments.

## 3. Phase Alignment System Design

**Figure 5:**
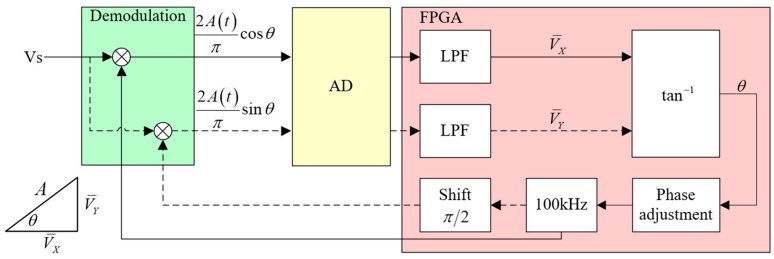
Principle of phase alignment system implemented on FPGA.

The block diagram of the phase alignment system is illustrated in Figure 5, employing the principle of a single-channel method for phase alignment.

In Figure 5′s block diagram, the FPGA generates a 100 kHz demodulation switch signal. This signal is then processed by the demodulation circuit to yield the output signal, as depicted in Equation (14). Subsequently, the signal is captured by AD and further processed within the FPGA. The FPGA proceeds to modify the phase of the demodulation switch signal, shifting it by π/2. At this point, the signal output from the demodulation circuit can be referenced using Equation (15). Once more, the signal is ultimately processed within the FPGA.

(14) $$V_{X}\left(t\right)=\frac{2A\left(t\right)}{\pi }\cos\theta$$

(15) $$V_{Y}\left(t\right)=\frac{2A\left(t\right)}{\pi }\sin\theta$$

In FPGA, the output signal of the demodulation module is processed by the LPF, which effectively filters out high frequency and periodic interference components, resulting in the extraction of the $\overset{-}{V_{X}}$ and $\overset{-}{V_{Y}}$ components. As shown in Figure 5, the phase difference θ between the AC induction signal and the demodulation switch signal is computed according to Equation (16).

(16) $$\theta =\tan^{-1}\frac{\overset{-}{V_{Y}}}{\overset{-}{V_{X}}}$$

Finally, the phase of the demodulation switch signal is adjusted in the phase adjustment module, so that the demodulation switch signal is aligned with the modulation signal.

**Figure 6:**
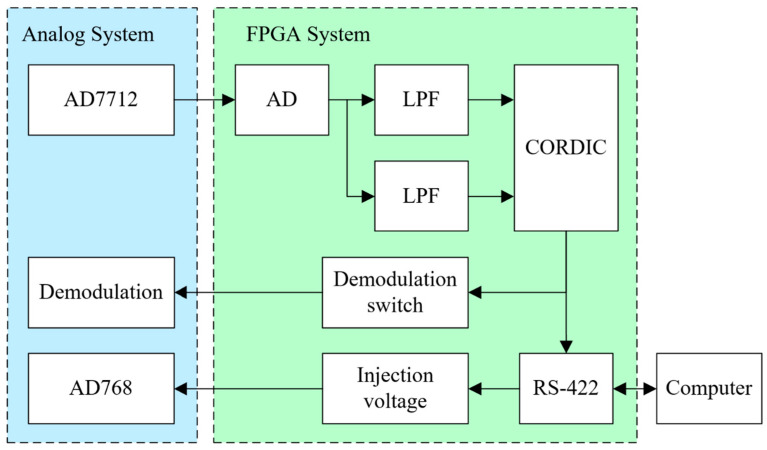
Phase alignment system software module in FPGA (including AD module, LPF module, injection voltage module, demodulation switch signal module, and RS-422 module).

The phase alignment system is implemented on the FPGA, as illustrated in the software system’s block diagram in Figure 6. The system can be broken down into various modules, including AD acquisition, LPF, CORDIC (coordinate rotation digital computer), demodulation switch signal generation, injection signal, and RS-422 modules.

### 3.1. AD Module

The primary role of the AD module is to convert the analog output from the demodulation circuit into a digital format for processing within the FPGA. This module utilizes the AD7712 chip, designed for low-frequency measurements, employing sigma-delta technology for high-precision 24-bit analog-to-digital conversion. Additionally, it incorporates on-chip registers to manage parameters like digital filter cutoff, input gain, signal polarity, and calibration modes.

### 3.2. LPF Module

The LPF module consists of an IIR module and a moving average filter. The IIR module, characterized by a feedback structure, exhibits favorable amplitude-frequency traits when phase characteristics are not taken into account. The filter adopts the Chebyshev II structure, whose order is M = 2, sampling frequency is $f_{s}$ = 10 Hz, passband frequency is $f_{pass}$ = 0.5 Hz, and stopband frequency is $f_{stop}$ = 5 Hz. In theory, the coefficient of the digital filter possesses infinite precision. However, in the FPGA, it is of finite length, necessitating quantification. There exists a certain deviation between the quantized coefficient and the original coefficient, which may cause the zero and pole deviation of the filter, thus preventing the attainment of the design’s intended performance. Therefore, 16-bit quantization is adopted in this paper.

In this paper, a moving average filter is employed to eliminate periodic interference. The principle involves storing consecutively collected *N* sample values in a fixed-length queue. Whenever a new data point is sampled, it is appended to the end of the queue, displacing the oldest data point. Subsequently, the *N* data points in the queue are averaged. This sliding filtering process serves to globally smooth the waveform. Following filtering, the $\overset{-}{V_{X}}$ and $\overset{-}{V_{Y}}$ components of the demodulated output are obtained, as indicated in Equation (17). Here, $V_{X,IIR}$ and $V_{Y,IIR}$ represent the outcomes of IIR processing on the demodulated output.

**Figure 7:**
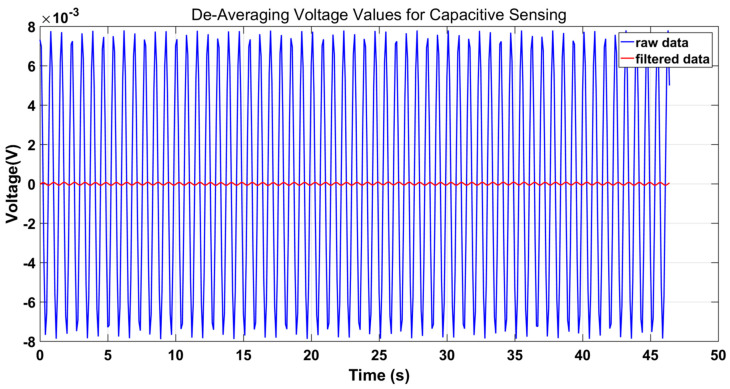
De-averaging the voltage values for LPF data (blue: capacitive sensing raw data; red: the filtered capacitive sensing data are used for phase alignment).

The filtering effect is visually demonstrated in Figure 7. The results clearly indicate that the adeptly designed LPF suppresses both periodic interference and high-frequency noise.

(17) $$\overset{-}{V_{X}}\left(t\right)=\frac{1}{N}\sum_{n=t-N}^{t}V_{X,IIR}\left(n\right)\;\overset{-}{V_{Y}}\left(t\right)=\frac{1}{N}\sum_{n=t-N}^{t}V_{Y,IIR}\left(n\right)$$

### 3.3. CORDIC Module

After the phase alignment system computes the $\overset{-}{V_{X}}$ and $\overset{-}{V_{Y}}$ components of the demodulation output, the phase difference can be computed according to Equation (13). The main method to implement arctan computation on FPGA is the CORDIC algorithm. The CORDIC module can realize the operation of trigonometric functions through the iterative rotation of the circular coordinate system [29], specifically as shown in Equation (18), which represents the rotation transformation of coordinate points.

(18) $$x_{i+1}=x_{i}-y_{i}\sin\varphi \;y_{i+1}=x_{i}+y_{i}\cos\varphi$$

The rotation angle of each iteration i is given in Equation (19), where $r_{i}$ = ±1, used to determine the rotation direction. And the scale factor K in Equation (20) is defined as the product of all $K_{i}$. The angle of each rotation is added up as shown in Equation (21).

(19) $$\varphi_{i}=r_{i}\tan^{-1}2^{-i}$$

(20) $$K_{n}=\prod_{i=0}^{n-1}K_{i}=\prod_{i=0}^{n-1}\sqrt{1+2^{-2i}}$$

(21) $$z_{i+1}=z_{i}-r_{i}\varphi_{i}$$

After *n* iterations of the initial coordinates $\left(x_{0},y_{0}\right)$, $\tan^{-1}y_{0}$ is computed by setting $x_{0}$ = 1 and $z_{0}$ = 0, as shown in Equation (22). Finally, $z_{n}$ is the result of the arctan.

(22) $$x_{n}\approx K\sqrt{x_{0}^{2}+y_{0}^{2}}\;y_{n}=0\;z_{n}\approx z_{0}+\tan^{-1}\left(y_{0}/x_{0}\right)$$

### 3.4. Demodulation Switch Signal Module and Injection Voltage Module

The demodulation switch signal module primarily generates a square wave signal at a frequency of 100 kHz, and derives the demodulation switch signal using the clock signal, $f_{clk}$, provided by the FPGA. The phase adjustment accuracy of the demodulation switch must exceed the computation accuracy of CORDIC, and the clock frequency is set to 410 MHz.

The injection voltage module is used to generate a 100 kHz injection signal with adjustable amplitude. It adopts DDS technology, which has the advantages of high frequency resolution, fast frequency change, and continuous phase. At the same time, the amplitude information of the injection signal can be adjusted online. In this paper, the AD768 chip is chosen as the digital-to-analog converter, and it is controlled by the FPGA to generate the injection voltage. The AD768 is a 16-bit high-speed digital-to-analog converter (DAC) known for its outstanding AC and DC performance. It provides a steady output of 100 kHz injection voltage, which fulfills the requirements of capacitive sensors.

### 3.5. RS-422 Module

This module primarily handles data transmission through the RS-422 serial port. The key information transmitted includes data collected by the demodulation module, phase deviation computed by CORDIC, amplitude information from the injection voltage module, and system status updates.

The meanings of symbols involved in this paper are summarized in Table 1.

## 4. Measurement Results and Discussion

### 4.1. Static Environment Testing

**Figure 8:**
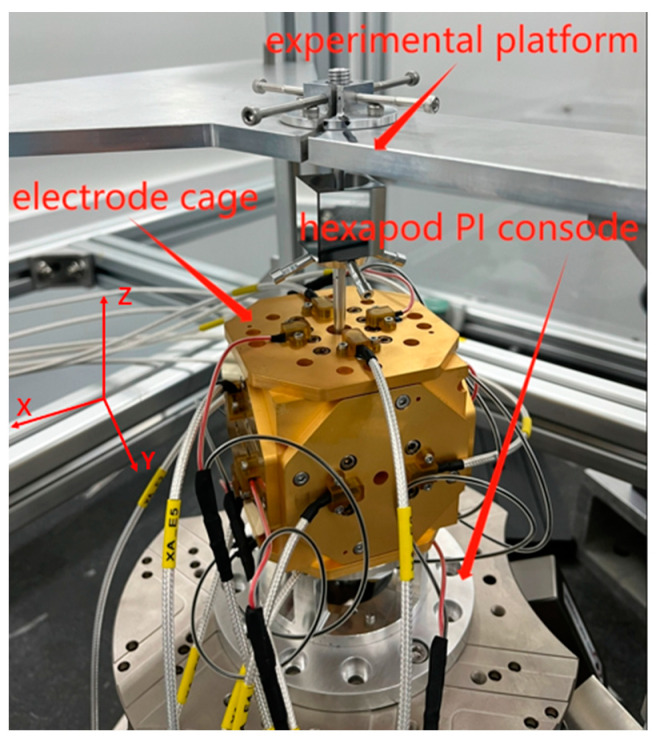
Phase alignment system test platform physical diagram.

The experimental test platform is established based on the configuration depicted in Figure 1. It employs capacitance sensing to measure the TM’s relative position. And this testing environment is depicted in Figure 8. Specifically, the TM is suspended and affixed onto the test platform, with the electrode cage affixed to the hexapod PI console. The hexapod PI console is kept static to simulate the static test environment.

**Figure 9:**
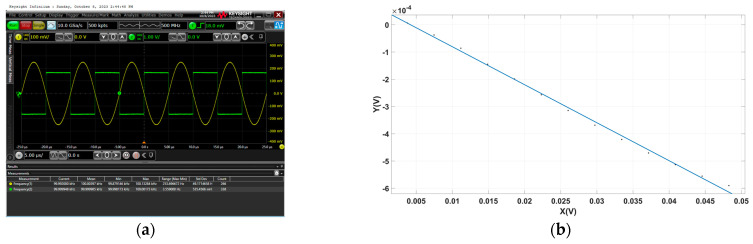
(**a**) The phase relationship between the demodulation switch signal and the AC induction signal is observed using an oscilloscope. (**b**) The fitting phase deviation of demodulation in the static environment.

Before the experiment begins, an AH2700A ultra-precision capacitance bridge is utilized to measure the input capacitance difference of the capacitance sensor on the *X*-axis, and the result is 0.085 pF. According to the research in [6], the capacitor sensor is in its linear operating range at this time. In the experiment, the phase alignment system proposed in this paper is employed to align the demodulation switch signal. Subsequently, the AC induction signal and the demodulation switch signal are observed through the oscilloscope. As depicted in Figure 9a, under static conditions, the system successfully achieves phase alignment between the AC induction signal and the demodulation switch signal.

According to the characteristics of the capacitive sensing, the amplitude of the AC induction signal is proportional to that of the injection voltage signal. Uniformly adjusting the amplitude of the injection voltage signal leads to an even adjustment of the modulation signal input, facilitating the measurement of both the X and Y components of the system. Finally, the phase deviation curve of the modulation circuit is fitted. As illustrated in Figure 9b, the phase deviation of the demodulation switch signal after phase alignment in the static environment is 0.01394 rad.

### 4.2. Dynamic Environment Testing

To simulate the effect of the suspended TM wobbling on the phase alignment of the demodulation switch signal during ground testing, a hexapod platform is employed to build a dynamic test environment. As depicted in Figure 8, the TM is both suspended and affixed onto the experimental platform, with the electrode cage firmly affixed to the hexapod PI console. The hexapod platform is moved along the *X*-axis to simulate a dynamic environment.

In the ground test, the length of the tungsten wire is l = 0.47 m, and the distance between the suspension point of the tungsten wire on the TM and its center of gravity is $l^{\prime }$ = 0.107 m. The gravitational constant is g = 9.8 $m/s^{2}$. According to Equation (13), the swing frequency of the TM is 0.66 Hz. To simulate this dynamic environment, the hexapod PI console moves sinusoidally along the *X*-axis at frequencies of 1.302 Hz, 0.977 Hz, 0.781 Hz, and 0.651 Hz. And the amplitudes of movement are 0.1 μm, 0.5 μm, 1 μm, 5 μm, and 10 μm, respectively.

In the simulated dynamic environment, this paper meticulously aligns the phase of the demodulation switch signal and uniformly adjusts the amplitude of the excitation signal to ensure a consistent input of the AC induction signal. After measuring the X and Y components of the system, the phase deviation curve of the demodulation circuit is accurately fitted. Table 2 presents the results of the calibration curve, unequivocally demonstrating the system’s capability to achieve precise phase alignment in dynamic environments, with an average phase deviation of 0.1395 rad. It is important to note that this experiment is conducted under ground test conditions, where the environmental temperature remains unregulated. Consequently, there may be slight fluctuations in the circuit performance metrics. Even within this uncontrolled environment, the system consistently maintains an average phase deviation of 0.1395 rad, meeting the current practical requirements. Furthermore, this study introduces and thoroughly analyzes a novel method for measuring phase deviation, marking the first comprehensive examination of this approach.

**Figure 10:**
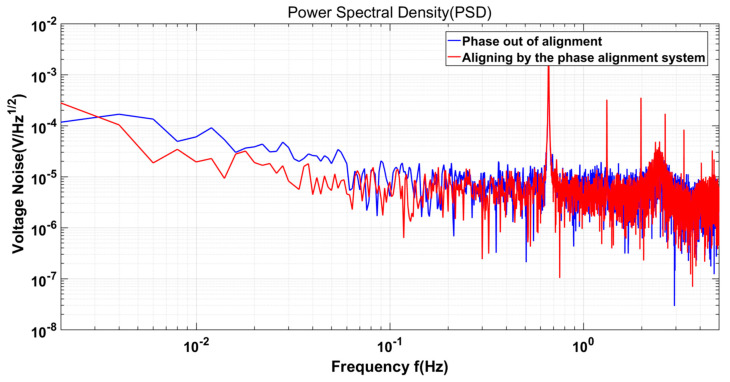
Voltage noise. The hexapod PI console platform has interference with the frequency of 0.66 Hz and the amplitude of 10 μm along the *X*-axis. Red: Capacitive sensing data are collected after phase alignment. Blue: The capacitance sensing data are collected without phase alignment. In the frequency band 10 mHz to 100 mHz, the noise of capacitive sensing is significantly reduced after phase alignment.

Meanwhile, the performance of the capacitive sensor processed by the phase alignment system is evaluated. Additionally, on the hexapod PI console platform simulating the dynamic environment, perturbations with a frequency of 0.66 Hz and an amplitude of 10 μm are applied along the *X*-axis. The phase alignment system performs phase alignment under the influence of dynamic disturbances, and then collects data from the capacitive sensor. In contrast, under the same interference conditions, the demodulation switch signal is not phase-aligned, and data from the capacitive sensor are collected. The voltage noise of a capacitive sensor is computed by taking the square root of the power spectral density (PSD) [30]. All data are processed using the LISA Technology Package for Data Analysis (LTPDA) toolbox [31]. As shown in Figure 10, within the range of 10 mHz to 100 mHz, the voltage noise of the phase alignment system is lower than that of the demodulation switch signal when the phase is not aligned. Specifically, at 10 mHz, the voltage noise after phase alignment is 19.42 $\mu V/\sqrt{Hz}$, which is significantly lower than the voltage noise of 60.19 $\mu V/\sqrt{Hz}$ without alignment. The results demonstrate that the phase alignment system proposed in this paper can effectively enhance the performance of capacitive sensors in the dynamic environment, filter out noise, and improve the SNR of the system.

## 5. Conclusions

This paper introduces a dynamic system’s demodulation switch signal phase alignment system. The system achieves precise phase alignment of the demodulation switch signal by adjusting its phase and computing the phase difference. The experimental results show that the system can accurately align the phase of the demodulation switch signal in static environments. Additionally, this paper employs a ground-based hexapod PI platform to simulate the conditions of the dynamic environment. It introduces a range of frequencies and amplitudes of interference along the *X*-axis. The results show that the average phase deviation is 0.1395 rad after using the system for phase alignment, indicating that the system can accurately align the demodulation switch signal phase in dynamic environments. At the same time, the voltage noise of capacitive sensing after phase alignment is significantly reduced, especially in the measurement band of capacitive sensing (10~100 mHz). The voltage noise at 10 mHz is 19.42 $\mu V/\sqrt{Hz}$, which is lower than that without phase alignment, indicating that the phase alignment system proposed in this paper can reduce voltage noise and improve the SNR of capacitive sensing. As the experimental environment lacks temperature control measures, the hardware circuit’s performance experiences fluctuations, affecting the phase alignment system to some extent. Subsequent enhancements to the experimental environment will, to some degree, bolster the performance of the phase alignment system. It is worth noting that the present experiment solely simulates phase alignment with a single degree of freedom. Future research will explore the effect of the phase alignment of crosstalk pairs between multiple channels in a multi-degree of freedom system. The phase alignment system proposed in this paper offers a precise means to align the demodulation switch signal’s phase within dynamic environments. This technology holds significant implications for future endeavors in inertial sensor technology for space applications.

## Figures and Tables

**Table 1:** The meaning of equation symbols in this paper.

Symbol	Meaning
$C_{0}$	Electrode capacitance for centered TM
$C_{1}$,$C_{2}$	Capacitance of two sensing electrodes
$\varepsilon_{r}{,\varepsilon }_{0}$	Vacuum permittivity and relative permittivity
S	The effective area of the electrode plate
d	The distance from the electrode to the TM
Δd	The displacement of the TM translation
ΔC	Differential capacitance
$C_{p}$	Sensing bridge tuning capacitance
L	Inductance
ω	Resonant frequency
$V_{inj}$	AC injection voltage signal
$V_{M}$	Injection voltage at the TM surface
$V_{BR}$	Voltage of the capacitive-inductive resonant bridge
$V_{s}\left(t\right)$	AC-induced signal
$V_{r}\left(t\right)$	The demodulation switch signal
$a_{0}$	The DC component of the Fourier series of the $V_{r}\left(t\right)$
$a_{m}$	The cosine component of the Fourier series of the $V_{r}\left(t\right)$
$b_{m}$	The sine component he Fourier series of the $V_{r}\left(t\right)$
$\omega_{0}$	The frequency of AC induction signal
θ	The phase difference between $V_{s}\left(t\right)$ and $V_{r}\left(t\right)$
A	The amplitude of AC induction signal
$f_{SW}$	The natural sway frequency of TM oscillation
l	The length of the tungsten wire
$l^{\prime }$	Distance from suspension point to TM center of gravity
$V_{X}$, $V_{Y}$	Demodulated X and Y components of the output
$V_{X,IIR}$, $V_{Y,IIR}$	The outcomes of IIR processing on the demodulated output
$\overset{-}{V_{X}}$, $\overset{-}{V_{Y}}$	Demodulation output after filtering out high-frequency interference
x, y	The coordinate point in CORDIC
r	The rotation direction in CORDIC
φ	The angle of each rotation in CORDIC
K	The scale factor in CORDIC
z	The sum of the angles of rotation in CORDIC

**Table 2:** Phase deviation in dynamic environments.

Interference Frequency (Hz)	Interference Amplitude (μm)	Phase Deviation (rad)
1.302	0.1	0.01477
	0.5	0.01556
	1	0.01198
	5	0.01265
	10	0.0149
0.977	0.1	0.00677
	0.5	0.01309
	1	0.01675
	5	0.01725
	10	0.01257
0.781	0.1	0.01504
	0.5	0.01532
	1	0.00918
	5	0.01725
	10	0.01400
0.651	0.1	0.01379
	0.5	0.01535
	1	0.01379
	5	0.01420
	10	0.01480
